# Epithelial-Mesenchymal Plasticity in Organotropism Metastasis and Tumor Immune Escape

**DOI:** 10.3390/jcm8050747

**Published:** 2019-05-25

**Authors:** Xiang Nan, Jiang Wang, Haowen Nikola Liu, Stephen T.C. Wong, Hong Zhao

**Affiliations:** 1 Center for Biomedical Engineering, University of Science and Technology of China, Hefei 230052, China; xnan2@houstonmethodist.org; 2 Department of Systems Medicine and Bioengineering, Houston Methodist Cancer Center, Weill Cornell Medicine, Houston, TX 77030, USA; hnliu@houstonmethodist.org; 3 Department of Orthopedics, Tongji Hospital, Wuhan 430050, China; wangjiangtjgk@163.com

**Keywords:** organotropism metastasis, EMT heterogeneity, tumor immune escape, cell–cell communication

## Abstract

Most cancer deaths are due to metastasis, and almost all cancers have their preferential metastatic organs, known as “organotropism metastasis”. Epithelial-mesenchymal plasticity has been described as heterogeneous and dynamic cellular differentiation states, supported by emerging experimental evidence from both molecular and morphological levels. Many molecular factors regulating epithelial-mesenchymal plasticity have tissue-specific and non-redundant properties. Reciprocally, cellular epithelial-mesenchymal plasticity contributes to shaping organ-specific pre-metastatic niche (PMN) including distinct local immune landscapes, mainly through secreted bioactive molecular factors. Here, we summarize recent progress on the involvement of tumor epithelial-mesenchymal plasticity in driving organotropic metastasis and regulating the function of different immune cells in organ-specific metastasis.

## 1. Introduction

The mechanisms of organotropism metastasis is one of the most unanswered questions in the field of cancer research. From the original “seed and soil” theory to recent discoveries on pre-metastatic niches (PMNs), our current understanding of organotropic metastasis is that this process is regulated by multi-facet factors including intrinsic properties of cancer cells, characteristics of organ microenvironments, and cancer cell‒organ interactions. Epithelial-mesenchymal transition (EMT) is recognized as an initial and critical event for the metastasis of carcinomas. Traditionally, tumor cells undergoing EMT lose their cell–cell adhesion and apico-basal polarity and gain the ability to migrate individually and invade basement membrane and blood vessels. Upon intravasation, these cells stay in the bloodstream as circulating tumor cells (CTCs) and evade immune attacks until extravasation at distant organs to seed micro-metastases. During seeding, they undergo the reverse EMT process, MET, to regain their epithelial characteristics and form secondary tumors or macro-metastases [1]. However, emerging studies identified the cellular plasticity of epithelial and mesenchymal state conversion of carcinoma cells during the metastasis process. Notably, tumor cells under partial EMT or hybrid EMT state, which means they keep both E and M properties, are likely to express or secret distinct bioactive factors and induce the formation of organ-specific PMNs; at seeding organs, the partial MET cells are more adaptive to the organ microenvironment and to forming colonization; these partial EMT and MET cells are more resistant to immune attacks by altering the function of different immune cells in systemic circulation and local organs. In this review, we focus on the heterogeneous EMT and MET phenotypes in primary and metastatic tumors, the contribution of partial EMT and MET cells in organotropism metastasis, their regulation of the function of immune cells, and mostly, the secreted molecular factors regulating the cell–cell interactions in organ-specific tumor microenvironments.

### 1.1. Organotropic Metastasis

Metastasis is a fatal step in cancer progression, 90% of patient mortality is due to complications from metastatic diseases rather than from primary tumors [2]. Tumor metastases to different organs is not a random process but is known to have organ-specific preference or “organotropism”. Organotropic metastasis remains the most intriguing but unanswered questions in cancer research. The “seed and soil” theory proposed by Steven first described site-specific metastasis [3]. Back to 1889, he proposed that metastatic tumor cells’ (“seed”) initiation of outgrowth in distant organs largely depends on crosstalk with the host microenvironment (“soil”). In the past several decades, extensive studies have enriched our understanding and indicate that organotropic metastasis is determined by multi-facet factors including cancer cells’ intrinsic properties (cancer subtypes or cancer cell subpopulations), the distinct organ microenvironment, and cancer cells‒organ interactions [4,5]. From the aspect of the intrinsic properties of cancer cells, for example, the hormone receptor positive (ER+/PR+) and human epidermal growth factor receptor 2 (HER2) positive (HER2+) breast cancer subtype has an especially high rate of bone metastases compared with other subtypes [6]. The triple-negative basal-like subtype is specifically associated with a low rate of bone and liver metastases but a high rate of brain metastasis [7]. At a genomics level, many exciting studies from Massagué’s group identified altered gene expressions that mediate metastasis in breast cancer, lung adenocarcinoma, and renal cell carcinoma to sites including the bone, lung, and brain [8,9,10,11,12,13]. From the aspect of the host microenvironment, different anatomical and histological characteristics of the host organs determine the ease with which cancer cells can invade and outgrow. For example, in bone marrow and liver, fenestrated sinusoidal endothelia permits the high permeability of tumor cells [14], while in the brain the blood–brain barrier (BBB), formed by tight conjunct endothelia, astrocytes, and pericytes, restricts the entry of many molecules and cells [15]. In addition, the chemical compositions and mechanical forces presented by the extracellular matrix (ECM) [16] and local stromal cell populations [17] have been recognized to play critical roles in organotropic metastasis. Furthermore, immune cells in both the host organ microenvironment and systemic circulation have close interactions with tumor cells and regulate organotropic metastasis [18]. 

The recent discovery of organ-specific PMNs in both preclinical models and clinical samples is a new paradigm for metastasis initiation and explicitly organotropic metastasis [19,20,21]. Before tumor cells metastasize, the formation of PMNs at distant targeted organs are induced mainly by tumor cell-secreted factors and tumor-shed extracellular vesicles (EVs) that alter the organ’s local milieu and create a tumor receptive microenvironment [19]. For example, PMNs consist of aberrant immune cells that are recruited from bone marrow [22,23,24]. Tumor cells seeding into PMNs get support to thrive and give rise to micro-metastasis; tumor cells seeding into non-PMN areas fail to form metastatic colonization. In contrast, specific niches, known as “sleepy niche” or dormant niches, also exist, in which disseminated tumor cells keep dormancy until the tissue homeostasis breaks and tumor cells awake to re-grow [19]. Because different sub-clones of tumor cells in one primary tumor can derive distinct and common secreted factors and Evs, and even one sub-clone of tumor cells can secret a variety of factors and Evs, multifocal PMNs in one organ and multiple PMNs in different organs can be formed, thus a primary tumor has the ability to metastasize to more than one organ and form polyclonal metastatic lesions within one organ. However, research in this field remains immature and there are many important questions that have not been elucidated, for example, studies on specific molecules expressed and/or shed by specific tumors to foster the formation of PMNs in specific organs are just emerging (see below section), thus this information is largely unknown; the dynamics of PMN formation has not been explored; and the contribution of other cellular components (such as adipocytes and sympathetic neurons) to PMN formation has not been explored [19]. Nonetheless, studying the molecular mechanisms of organotropism metastasis is critical, not only for biomarker-based prediction and prognosis, but also for the development of innovative therapeutic strategy, and the eventual prevention of cancer metastasis. 

### 1.2. Heterogeneous EMT Phenotypes and the Activation/Regulation Complexity

Epithelial-mesenchymal plasticity is mostly referred to as the different cellular states when cells are undergoing EMT and its reverse program MET and intermediate states between these two, partial EMT or hybrid EMT. EMT is strictly defined as cell morphological changes from epithelioid to mesenchymal/fibroblastoid/spindle-shape and is accompanied by drastic and persistent molecular changes [25]. It has been accepted as a critical program allowing stationary epithelial cells to gain motility in order to migrate and invade during embryogenesis, organ development, tissue regeneration, and organ fibrosis. The activation of the EMT program has been implicated in cancer initiation, invasion, metastasis, and chemo-resistance as demonstrated by extensive studies in the past decade [26,27,28,29]. However, it is different from the context of wound healing and embryonic development where the intermediate states of EMT are distinct and well-studied, while in cancer, EMT and MET are not “all-or-none” processes. Cancer cells co-expressing both canonical epithelial and mesenchymal markers are stable over multiple passages and metastable [30]. Cancer EMT progression is a multi-dimensional nonlinear process and EMT and MET are not binary processes [30]. 

EMT primarily encompasses a cell morphological change. By performing intravital two-photon microscope imaging, our group was able to track and analyze individual EMT tumor cells in red (E)-to-green (M) fluorescent color switching mouse breast tumors [31]. Per the emergence of green (M) fluorescent cells in live mouse tumors, the cells’ axial ratios and moving distances, we quantitatively identified the heterogeneous sub-populations of the EMT cells at different tumor stages, i.e., fibroblast-like EMT cells, migratory EMT cells, and quiescent EMT cells. For example, in the early-stage small tumors (~2 mm diameter), the fibroblast-like EMT cells constituted about 5% of all of the EMT cells. These cells were recognized by their spindle-like shape and long linear processes tightly attached to the ECM, but they do not have high migratory ability. About 50% of the EMT cells exhibited tropism movement and iterative elongation and contraction of the pseudopods toward one main direction; we defined them as migratory EMT cells. Another 20% of the green (M) cells kept an amoeboid appearance without pseudopods and were almost static without obvious shape changes during the 4–6 hours imaging period. These cells are mostly located in the surrounding of the migratory EMT cells, and we defined them as temporarily quiescent EMT cells [31]. The migratory EMT cells were characterized as losing cell polarity, acting like amoeba, and migrating towards stimuli, which may mainly contribute to metastasis [32]. The fibroblast-like EMT cells kept partial polarity and attached tightly to the ECM but without movement, which may develop into cancer associated fibroblasts [33]. In addition, a significant percentage (~20%) of EMT cells were of quiescent subtype, and we did not observe any changeovers between the quiescent and migratory EMT cells in the 4–6 hours imaging period. However, most of the migratory and quiescent EMT cells locate relatively closely (<50 um), but far apart from the clusters of fibroblast-like EMT cells (>100 um), which made us postulate that the migratory and quiescent EMT cells might have paracrine interplays or autocrine signals to maintain their equilibrium and give rise to metastasis [31]. Further characterization of the molecular composition of each subtype and delineation of their evolution or transformation is undergoing.

Aiello et al. also found the existence of divergent EMT programs in different cancer types [8]. In mouse pancreatic ductal adenocarcinoma (PDAC), the well-differentiated tumors are associated with a persistence of E-cadherin (ECAD) mRNA and a re-localization of ECAD protein inside the cells, which is termed as a partial EMT program (P-EMT). In contrast, the poorly-differentiated tumors tend to undergo a complete EMT (C-EMT) with losing ECAD mRNA and protein expression [34]. By cross-referencing the P-EMT and C-EMT gene signatures to the expression data from the Cancer Cell Line Encyclopedia (CCLE), several human pancreatic cancer cell lines were stratified as P-EMT or C-EMT. Similarly, basal-like breast cancer cells were characterized as C-EMT, but luminal A, B, or normal-like breast cancer cells were associated with P-EMT [34]. Puram et al. [35] profiled single cell transcriptome from matched pairs of primary tumors and lymph node metastases in head and neck cancer patients. They identified that cells expressing the P-EMT program spatially localized to the leading edge of primary tumors in proximity to cancer-associated fibroblasts (CAFs), and predict lymph node metastases. 

Functionally, our studies and others found that many carcinoma cells may metastasize without completely losing the E (epithalial) and/or attaining the M (mesenchymal) traits [36,37]. Cells in the hybrid E/M phenotype keep both E and M traits, migrating collectively as commonly seen in the multicellular migration in ECM [31] and CTC clusters [38,39]. By examining the cell invasion and migration properties of the above mentioned histological-relevant EMT programs in PDAC, it was found that in the C-EMT tumorspheres, spindle-like protrusions of single cells at the edges of the primary cell mass were primarily observed. By contrast, in P-EMT spheres both budding cell clusters as a collective group and single cells escaped from the primary cell mass [34]. Furthermore, >95% of the CTCs in the C-EMT cell line-derived PDAC models were present as single cells, while >50% of CTCs existed as tumor cell clusters in the P-EMT cell line-derived models [34,40]. The single CTCs from the C-EMT tumors lacked staining of ECAD protein, and tumor cell clusters arising from P-EMT tumors retained ECAD staining only at the cell-cell contact points but not on the cluster surface [34]. The CTC cluster cells are resistant to anoikis, and they extravasate the vessels more efficiently and are 50 times more metastatic than individual CTCs [41,42]. Therefore, the P-EMT program poses a higher metastatic risk than the C-EMT program in cancer patients [43]. At the metastatic organs, heterogeneous MET phenotypes are also reported. Although metastatic carcinomas commonly express epithelial markers, mesenchymal markers are often examined in patients. For example, in the brain microenvironment, metastatic lung cancer cells showed increased expression of the epithelial marker ECAD as well as elevated levels of transcription factor ZEB1 and mesenchymal markers VIM [44,45], reflecting the partial EMT/MET phenotype. Recent studies revealed the existence of both MET-dependent and MET-independent metastasis, i.e., a MET-dependent metastasis in carcinosarcomas and a MET-independent metastasis in prostate cancer [46]. The traditional EMT “master” transcription factors (EMT-TFs) and miRNAs which maintain the epithelial phenotype mainly regulate the MET-dependent metastatic mechanisms [46]. 

In the complex process from primary tumor to metastasis, cancer cells adaptively change in the hostile environment by transitioning back-and-forth from differentiated to undifferentiated or partial EMT phenotypes [28,47]. The phenotypic plasticity of EMT subtypes is mainly regulated by functionally pleiotropic EMT-TFs and miRNAs [48,49]. Epithelial cell markers are transcriptionally repressed through the action of EMT-TFs. In parallel, mesenchymal markers are induced to express [50]. Furthermore, the EMT-TFs guide the recruitment of epigenetic machinery to the chromatin, thus allowing the proper regulation of gene expression [51,52]. For example, the E-cadherin promoter is regulated epigenetically via methylation in most intra-ductal breast carcinomas, thus E-cadherin expression is dynamically modulated by the microenvironment [53]. In addition, recent studies revealed post-transcriptional regulation of EMT activation. Studies on PDACs showed that C-EMT tumor cells lost both membranous and intracellular ECAD consistent with the loss of Ecad mRNA. By contrast, P-EMT cells store epithelial proteins (ECAD, β-catenin, Claudin-7 and EpCAM) intracellularly and re-locate them back to the cell surface through recycling endocytic vesicles [34]. Tumor microenvironment factors always activate EMT through multiple mechanisms. For example, under hypoxia the elevated hypoxia induced factor-1 (HIF-1) can bind to the promoter region of EMT-TFs and regulate their expressions [54]. In addition, inflammatory cells including neutrophils, lymphocytes, macrophages, and myeloid-derived suppressor cells (MDSCs), which secrete inflammatory cytokines stimulated by hypoxic stress, including tumor necrosis factor α, transforming growth factor β (TGF-β), interleukin 1 (IL-1), IL-6, and IL-8, all contribute to hypoxia-induced EMT [54,55,56]. For the P-EMT or E/M hybrid state, phenotypic stability factors (PSFs) including OVOL and GRHL2 have been characterized in stabilizing such EMT state [57], and OVOL by coupling with miR200/ZEB/LIN28/let-7 circuit has been examined to increase the stemness of the hybrid E/M phenotype [58,59]. 

## 2. Epithelial-Mesenchymal Plasticity and Cancer Organotropism Metastasis

A new mechanism revealed how epithelial/mesenchymal plasticity determines PDAC metastasizing to lung and liver [40,60]. The authors found that the expression of intact p120 Catenin (P120CTN), a protein that binds and stabilizes ECAD, appeared predominantly in liver metastasis of the PDAC mice; however, genetic abrogation of P120CTN significantly shifts the metastatic burden to the lungs [60]. This striking organotropism change is mediated by the differential epithelial status of tumor cells, i.e., invasive tumor cells in the primary tumor showed low E-cadherin expression but regained in liver metastatic lesions; in contrast, tumor cells in the lung metastases lacked expression of P120CTN or E-cadherin, suggesting the occurrence of MET in liver metastasis, but lung metastatic cells remained at the M state. This conclusion was further verified by an experiment that directly monitored the tumor cell colonization in liver and lung [60]. Cells with wild-type or single copy P120CTN, but not bi-allelic deletion, which kept the ability to stabilize ECAD and convert tumor cells to E state, are able to form liver metastases. However, PDAC cells with bi-allelic deletion of P120CTN lost the ability to stabilize ECAD and undergo MET, bypassed the liver, and preferentially went to the lung. The authors concluded that P120CTN modulated epithelial plasticity and liver or lung organotropic metastases in PDAC [60].

Although other mechanisms directly connecting EMT plasticity with organotropism metastasis are lacking, emerging evidence indicate that epithelial plasticity regulates cancer stemness [61], for which cancer stem cells (CSCs) are responsible for organotropism metastasis [62,63,64]. In certain studies inhibition of EMT has been reported to promote cancer stemness and is associated with tumor-initiating for metastatic colonization. However, activation of EMT was also shown to inhibit stem-like property [61]. MET has been noted to promote cancer stemness. For example, inhibitor of differentiation 1 (Id1) induces MET and stemness in breast cancer cells by antagonizing transcriptional factor Twist1 [65], and transient expression of Twist1 promotes the coexistence of both epithelial and mesenchymal features in the cells [66]. Existence of partial EMT/MET cells provides a reasonable explanation for this conflicting evidence, indicating that the ‘intermediate state’ of cancer cells may be more flexible in cell invasion and regulation of stem-like properties, especially when considering the temporal dynamics of the metastasis process in vivo. There are many observations to support this statement. For example, CTCs have been shown to express both epithelial and mesenchymal markers [67], and patients with advanced metastatic cancer have a high frequency of partial EMT/MET CTCs [39]. Furthermore, the partial EMT/MET cells in primary ovarian cancer and prostate cancer showed higher self-renewal and tumor-initiating ability [68,69]. Beerling et al. [70], tracked the ECAD^high^ epithelial and ECAD^low^ mesenchymal tumor cells in liver metastasis of PyMT-MMTV mouse breast tumors. They found that although intrinsically the epithelial and mesenchymal cells differ in stemness, this difference does not provide a significant metastatic outgrowth advantage because mesenchymal cells adapt an epithelial state after the first few cell divisions. This study further indicates the complex EMT plasticity in in vivo tumor metastasis. miRNAs were studied extensively in mediating the regulations of EMT/MET plasticity and stemness. miR-200 families were shown to promote MET, which also increases metastatic colonization in breast cancer [71]. miR-30 family members inhibited EMT through TWF1 and inhibited CSC-mediated lung metastasis [72]. miR-7 suppresses brain metastasis of breast cancer CSC by modulating KLF4 [73].

There are many more studies exploring miRNAs in cancer metastasis, and miRNAs in EMT regulation, thus we summarized here some speculations linking organ-specific EMT with metastasis initiation through miRNAs. For example, skeletons are the organ most affected by various metastatic cancer cells. Almost all important EMT regulators have been identified in the bone microenvironment facilitating bone metastasis formation, including hypoxia, various growth factors (TGF-β, epithelial growth factors, vascular endothelial growth factor, insulin-like growth factors, platelet-derived growth factor, and parathyroid hormone-related protein), cytokines (IL-1, 6, 8, 11), and other signaling molecules, including integrins, matrix metalloproteinases (MMPs), notch, Wnt, hedgehog signaling, and bone morphogenetic proteins (BMP) signaling pathways [74]. 

We speculate that miRNAs target host stroma in regulating organotropic metastasis by affecting tumor cell EMT. For example, breast cancer-secreted miR-122 promotes tumor metastasis to the brain and lungs by reprogramming glucose metabolism in the PMNs [75]. This process is likely accompanied by activated EMT in tumor cells [76]. Expression of miR-23b/27b/24 cluster promotes breast cancer lung metastasis by targeting metastasis-suppressive gene prosaposin [77]; these miRNAs also promote TGF-β1-induced EMT by directly targeting CDH1 and activating Wnt/β-catenin signaling [78,79]. Recently, Schirijver et al. compared global miRNAs expression in primary breast tumors and matched multiple distant metastases. miR-106b-5p was found to be an independent predictor of lung and gastrointestinal metastases, and miR-7-5p and miR-1273g-3p can predict skin and ovarian metastases, respectively [80]. These miRNAs have all been experimentally validated to regulate the EMT phenotypes of tumor cells [81,82,83].

Exosomes carrying specific miRNAs are recognized to not only function as vehicles to promote organ-specific metastasis but also mediate EMT regulation. Metastatic breast cancer cell-secreted miR-105 was shown to be transferred in exosome to endothelial cells and destroyed vascular endothelial barriers by targeting the tight junction protein Zonula occludens (ZO-1). This process was verified in experimental settings in promoting lung and brain metastasis [84]. Zhang et al. reported that brain astrocyte-derived exosomes promoted brain metastatic tumor growth from breast and lung cancer by transferring PTEN-targeting miR-19a to these cancer cells [85], and miR-19a has been well reported as an EMT promoting miRNA in lung cancer [86]. Tumor exosomes are shown to educate selected host tissues toward a prometastatic phenotype. In the rat pancreatic adenocarcinoma model ASML with preferential draining lymph nodes and lung metastasis, tumor exosomes and the exosomal mRNA and miRNA are taken up and recovered by lymph node stroma cells and lung fibroblasts after subcutaneous injection [87]. While the mRNAs’ translation was barely detected in the target cells, the miRNAs profoundly affected the transcriptome of these cells. Remarkably, exosomal miR-494 and miR-542-3p suppressed the expression of cadherin-17, up-regulated the MMPs transcription, and prepared a pre-metastatic niche for the lymph node and lung metastasis [87]. Both miR-494 and miR-542-3p have been demonstrated as inhibitory factors for EMT in pancreatic cancer and other cancer types [88,89].

In addition to exosomal miRNAs, Lyden et al. demonstrated that the exosomes released from human lung-, liver- and brain-tropic tumor cells preferentially fuse with resident cells at their predicted destination, i.e., lung fibroblasts and epithelial cells, liver Kupffer cells, and brain endothelial cells [90]. These exosomes mediated tumor cell and organ cell interaction in the organotropic metastatic niche. The authors observed that treatment with exosomes from lung-tropic models redirected the metastasis of bone-tropic tumor cells to lung [90]. The distinct role of different exosomal integrins in the organotropic metastases was further elucidated, e.g., exosomal integrin αvβ5 in breast cancer cells specifically binds to Kupffer cells in facilitating liver metastasis, whereas exosomal integrins α6β4 and α6β1 bind lung fibroblasts and epithelial cells, facilitating lung metastasis [90]. Integrins comprise heterodimer ECM receptors that are essential in enabling tumor cells to interact with ECM remodeling in the initiation and progression of EMT [91]. Different integrins engage with different ECM components, i.e., collagen type IV (α1β1, α2β1), laminins (α3β1, α6β1), fibrillin (α5β1, αVβ3, αVβ6), perlecan, and versican (β1) [92]. Some are also associated with ECAD that are required for EMT progression by integrating the TGFβ and β-catenin signaling [91]. In addition, changes of the integrin repertoire during EMT correlate with the increased expression of proteases, such as MMP2 and MMP9, enhancing ECM protein degradation and enabling invasion [91]. 

## 3. Epithelial-Mesenchymal Plasticity and Tumor Immune Escape in Metastatic Organs

**Figure 1:**
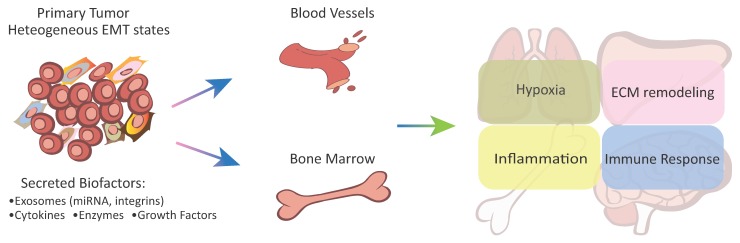
Epithelial-mesenchymal plasticity of carcinoma cells plays key roles in regulating organ microenvironment and local immune landscape in leading organotropism metastasis. Primary carcinoma cells under heterogeneous EMT states produce and secret a variety of bioactive factors, including exosomes carrying specific miRNAs, integrins, inflammatory cytokines, growth factors, and extracellular matrix enzymes to induce PMNs at distant organs. These bioactive factors mainly regulate microenvironmental hypoxia, inflammatory, ECM remodeling, and immune cell function.

Clinical achievements of cancer immunotherapy are currently outpacing our scientific understanding of the immune-related mechanisms for organotropic metastasis. Different factors in regulating the sensitivity of organ-specific metastases versus primary tumors to immunomodulation remain understudied. However, the heterogeneity of tumor immune landscapes both locally and systemically [93] could be partly attributed to the tumor epithelial-mesenchymal plasticity in modulating antitumor immunity from tumor microenvironment components [94] (Figure 1). 

Bone and the immune system are strictly linked to each other because all immune system cells are derived from hematopoietic stem cells that reside in bone, and many immunoregulatory cytokines influence the fate of bone cells. Moreover, many cytokines and secreted factors from immune and bone cells promote tumor growth in bone, contributing to the vicious cycle of bone metastasis [95]. As we mentioned before, almost all bone microenvironment factors are involved in regulating tumor EMT states [74]. The interactions between T cells and osteoclast precursors through reciprocal CD137/CD137L and RANK/RANKL regulate bone absorption in bone metastasis [96]; RANK/RANKL induces EMT in breast cancer [97]. Since MDSCs are progenitors of the osteoclast precursors, it is not surprising that they are largely increased in bone metastatic patients. MDSCs themselves could enhance tumor growth in bone through accumulating in secondary lymphoid organs and leading to a strong inhibition of the antitumor T cell response [95]. The accumulation of MDSCs in secondary lymphoid organs is mediated by the Wnt/β-catenin pathway [98], which is also an important EMT regulator. MDSCs have also been implicated in MET in lung metastasis. In the lung PMN of MMTV-PyMT breast tumor mice, accumulated MDSCs secrete versican, an extracellular matrix proteoglycan. Versican stimulated MET of metastatic tumor cells by attenuating phospho-Smad2 levels, which resulted in elevated cell proliferation and accelerated metastases [99]. As a primary tumor grows and becomes more hypoxic and inflammatory, tumor cells secret factors and extracellular vesicles [90,100] to attract MDSCs from bone marrow, initiating the pre-metastatic niche. The distant organ microenvironment is also adapted by these tumor secreted factors to accept the bone marrow derived cells and CTCs, thereby being shaped into a tumor-promoting metastatic niche characterized by increased angiogenesis and vascular permeability, ECM remodeling, chronic inflammation, and immunosuppression [21,101].

In brain metastasis, the STAT3-positive reactive astrocytes not only suppressed the activation of CD8+ T cells, but also promoted the expansion of CD74+ microglial/macrophages, which produces tumor growth promoting factors, thereby benefiting metastatic tumor growth in brain [102]. In patients, blocking STAT3 signaling in reactive astrocytes reduces experimental brain metastasis from different primary tumor sources, even at advanced stages of colonization [102]. STAT3 has long been recognized as a key stimulator of EMT in carcinoma [103], and recent studies revealed a EMT-like process in reactive astrocytes in primary brain tumors [104]. The increased expression of EMT-related factors in brain metastasis was found not only in tumor cells, but also in tumor-associated astrocytes [105].

Involvement of other immune cells in organ-specific metastasis have been explored in recent years as reviewed in [106], including metastasis-associated macrophages, neutrophils, natural killer (NK) cells, and T cells. Secreted factors from both tumor cells and stromal cells are the key factors controlling the functions of these immune cells, and again, many of them also regulate tumor epithelial-mesenchymal plasticity.

### 3.1. Metastasis-Associated Macrophages

Macrophages have been shown to promote lymph node, lung, and brain metastasis in breast cancer. Piao et al. reported that triple-negative breast cancer cell-derived exosomes induced M2 polarization of macrophages that created favorable conditions for lymph node metastasis, although the exact signaling factors in the exosomes were not characterized [107]. In the study by Linde et al. CD206^hi^ intra-epithelial macrophages in the very early stage of mammary intra-epithelial neoplasia in mice, which is similar to ductal carcinoma in situ (DCIS) in humans, were shown to respond to tumor secreted chemokine ligand 2 (CCL-2), which in turn stimulates macrophages to produce Wnt-1, leading to disruption of E-cadherin junctions between early cancer cells and propelling lung dissemination. Transient depletion of macrophages in mice at the “DCIS” stage reduced lung metastatic burden later in mice life [108]. In addition, in breast cancer lung metastasis mouse models, CCL-2 secreted by both tumor cells and endothelial cells preferentially recruited C-C chemokine receptor type 2 (CCR2+) macrophages to lungs, resulting in increased metastatic seeding and tumor outgrowth [109]. Anti-CCL2 treatment in these mice showed good efficacy, and discontinuation of anti-CCL2 treatment increased lung metastasis and accelerates mice death [110]. CCL-2 also has also been shown to play a detrimental role in brain metastasis. Zhang et al. demonstrated that breast cancer cells secreted large amounts of CCL-2 in vivo when infiltrating the brain parenchyma, resulting in the recruitment of IBA1+ macrophages that reciprocally enhance the metastatic outgrowth [85]. EMT program has been reported to stimulate the production of proinflammatory factors by cancer cells including CCL-2 [111], and CCL-2 specifically has been demonstrated to induce EMT in cancer cells [112]. 

### 3.2. Metastasis-Associated Neutrophils

The role of neutrophils has been debated on both promoting and inhibiting metastasis [113]. Recent studies indicate that depletion of neutrophils inhibited lung metastasis, and the iron-transporting protein transferrin was identified as the major mitogen for tumor cells secreted by neutrophils [114]. Granulocyte-macrophage colony-stimulating factor (GM-CSF), which is produced primarily by tumor cells, is a selective inducer of de novo transferrin synthesis in neutrophils through the Jak/Stat5β pathway [114]. Interestingly, cancer cells that express the GM-CSF receptor may undergo EMT through the GM-CSF autocrine mechanism [115], and mesenchymal cells differentially secrete GM-CSF [116]. Neutrophils are the most abundant circulating immune cell population. They were shown to escort CTCs (form CTC-neutrophil clusters) and enable cell cycle progression in disseminated tumor cells [117]. Such CTC–neutrophil clusters represent the most efficient metastasis-forming cell subpopulation in breast cancer CTCs, and their presence in the patients’ bloodstream is associated with a poor prognosis [117]. Vascular cell adhesion molecule 1 (VCAM1) was identified as the functional mediator for CTC-neutrophil interaction [117]. Although no difference on EMT-related genes was found between the CTCs with or without neutrophil escort, CTCs in general have been linked with C-EMT or P-EMT, as we discussed in the first section of this review. Intriguingly, VCAM-1 over-expression in normal breast epithelial cells controls the EMT program and has been associated with poor clinical prognosis in breast cancer patients [118].

### 3.3. Metastasis-Associated Natural Killer (NK) Cells

There is a general consensus that NK cells exert cytotoxicity against metastatic tumor cells. EMT activation in tumor cells during metastasis cascade is also accompanied by altered cell-surface ligands recognizable by NK cell-activating receptors, thus increasing susceptibility to NK cells [119,120]. A recent study by Chockley et al. reported that NK cells were activated to attack metastatic EMT tumor cells through the balance of activating and inhibitory receptors engaged by different ligands, and the EMT induced NK cell activity mediated immunosurveillance in lung metastasis [120]. Specifically, NK cells express killer lectin-like receptor G1 (KLRG1), which is an inhibitory receptor, and E-cad is an inhibitory ligand that engages KLRG1. The down-regulated E-cad during EMT released its inhibitory effect on KLRG1 and led to the activation of NK cells. Meanwhile, EMT also induced expression of cell adhesion molecule 1 (CADM1), which is an activating NK ligand and binds to the cytotoxic and regulatory T cell-associated molecule (CRTAM) receptor on NK cells. CADM1 is identified as a tumor suppressor and is frequently down-regulated in various types of tumors. Depletion of NK cells allowed spontaneous metastasis without affecting primary tumor growth in lung cancer [120]. 

### 3.4. Metastasis-Associated T Cells

T cell infiltration is crucial to tumor microenvironments and has been extensively studied in primary tumors [121]. However, T cell-dependent mechanisms involved in organ-specific metastasis remain underexplored. Mansfield et al. studied the T-cell clonal evolution in primary non-small cell lung tumors (NSCLC) and paired brain metastases [122]. They found significantly less numbers of unique T cell clones in brain metastases than those in primary tumors, and the clones were minimally overlapped, suggesting a divergent tumor immunogenicity following metastasis [122]. However, despite the contraction in the number of T cell clones, brain metastases harbored higher non-synonymous mutation burdens than primary lesions which may lead to the emergent expression of neoantigens [122]. Thereby, clinical response to anti-programmed cell death-1 (PD-1) monotherapy with pembrolizumab has shown intracranial response rates of 20–30% in patients with NSCLC or melanoma brain metastases [123]. The combination of nivolumab and ipilimumab (anti-PD-1 and anti-cytotoxic T-lymphocyte-associated protein 4 (CTLA-4)) showed an intracranial response rate of 55% in patients with melanoma brain metastases [124]. The emergence of neoantigens in brain metastatic tumors may be related to the very active neurogenesis, cellular differentiation and reprogramming state as evidenced by the co-expression of the epithelial marker with the mesenchymal marker and the high expression of stem cell markers [45].

## 4. Conclusions

**Figure 2:**
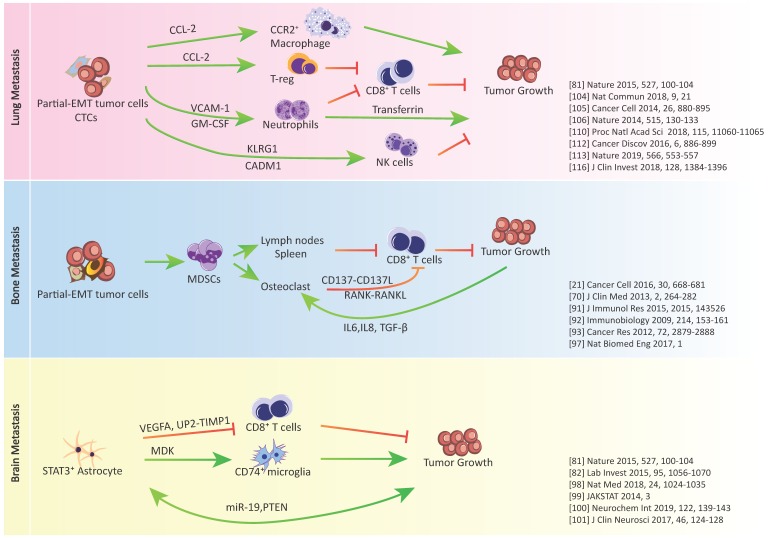
Crosstalk between cancer cells, immunosuppressive cells and immune effector cells in lung, bone, and brain metastasis.

In solid tumors, of which 90% are epithelial in nature, epithelial-mesenchymal plasticity is a fundamental factor in governing metastasis. As shown in Figure 2, emerging data have shown that certain types of tumors with heterogeneous EMT states or different degrees of EMT are prone to metastasize to different organs. Although the underlying mechanisms remain to be explored, the current studies indicate that cellular plasticity is linked with constant changes to produce various bioactive factors. The secreted bioactive factors not only contribute to shaping PMNs at specific organ sites, but also modify the local immune landscape, and in the meantime increase the plasticity of the niche cells. The niche cells reciprocally produce bioactive factors and interact with tumor cells and among themselves, leading to organotropism metastatic tumor growth. Thus, systematic studies of cell–cell communication on organ-specific tumor metastasis models will enable researchers to have a more precise picture of the co-evolution of metastatic tumor cells and their surrounding microenvironment, and offers new ways for therapeutic exploitation.
